# Certainty in Categorical Judgment of Size

**DOI:** 10.1371/journal.pone.0006198

**Published:** 2009-07-10

**Authors:** Eric J. Fimbel, René Michaud, Mathieu Martin

**Affiliations:** 1 Fatronik Foundation Research Center, Donostia, Spain; 2 Institut universitaire de gériatrie de Montréal, Montréal, Canada; 3 École de technologie supérieure, Montréal, Canada; University of Leuven, Belgium

## Abstract

The certainty of judgment (or self-confidence) has been traditionally studied in relation with the accuracy. However, from an observer's viewpoint, certainty may be more closely related to the consistency of judgment than to its accuracy: consistent judgments are *objectively certain* in the sense that any external observer can rely on these judgments to happen. The regions of certain vs. uncertain judgment were determined in a categorical rating experiment. The participants rated the size of visual objects on a 5-point scale. There was no feedback so that there were no constraints of accuracy. Individual data was examined, and the ratings were characterized by their frequency distributions (or *categories*). The main result was that the individual categories always presented a *core of certainty* where judgment was totally consistent, and large *peripheries* where judgment was inconsistent. In addition, the geometry of cores and boundaries exhibited several phenomena compatible with the literature on visual categorical judgment. The ubiquitous presence of cores in absence of accuracy constraints provided insights about objective certainty that may complement the literature on subjective certainty (self-confidence) and the accuracy of judgment.

## Introduction

### Certainty in judgment of size

When we describe the size of an object by means of words like ‘small’, ‘large’, ‘huge’, we may be more or less *certain* about the size and/or about the word to use. Certainty is a key factor when different courses of action stem from judgment. For instance suppose we want to grasp a stone. If we judge that it is ‘tiny’, we will pick it with two fingers, we will use one hand if it looks ‘medium-sized’ and two hands if it looks ‘large’. If our judgment is certain, we may act immediately. Otherwise, we may inspect the stone more carefully, take more time to consider, and eventually use a trial-and-error strategy.

In one of its meanings, *Certainty* is subjective. *Feeling certain* means “firmly believing, having no doubts” [1]. Subjective certainty (or *self-confidence*) is an individual feeling about our own judgment. In laboratory experiments, self-confidence may be expressed or rated post-hoc [2]. There is no straightforward relationship between self-confidence and accuracy. In fact, for general knowledge tasks, the relationship is inverse (*hard-easy effect*), although feedback may reduce this mismatch [3]. Errors may affect self-confidence in different ways. It was proposed that Brunswikian errors (lack of knowledge) elicit over-confidence in cognitive judgment and Thurstonian errors (perceptual noise) under-confidence in perceptual judgment [4]. However, this dichotomy has been experimentally challenged [5]. In categorical judgment, confidence depends on the categories, natural vs. artificial [6]. Conversely, the lack of confidence increases the gradedness of the responses thus the observed categories look less crisp [6]. The lack of straightforward relationship between self-confidence and the judgment itself is not surprising, given the existence of an epistemological gap: judgment is observable whereas self-confidence, like consciousness is an individual state of mind, unreachable by observation.

However, in another of its meanings, certainty is objective. *Something certain* means “something that you can rely on to happen” [1]. For instance, the phases of the moon and the tides are objectively certain. Objective certainty depends on the phenomenon and/or its causes, not on the observer. In the case of judgment, objective certainty is *not* the subjective feeling of the judge: it is the certainty shared by external observers that a judgment will occur under given circumstances. Objective certainty of judgment is thus directly determined by its predictability and its *repeatability*, i.e., under the same circumstances, the judgment will be the same. In real life, repeatability may remain an abstraction. However, in laboratory experiments, the observable equivalent of repeatability is the *consistency* (i.e., “something that always happens in the same way” [1]). Consistency is a graded variable that can be measured as soon as judgment is reproduced in “identical circumstances”. We can thus determine objective certainty experimentally, as *absolute* consistency.

This led us to design an experiment to study the consistency of judgment. Because objective certainty, like subjective certainty, is independent from truth and/or accuracy, we studied consistency independently from accuracy, i.e., in a task in which there were no accuracy constraints. Stimuli of different size were presented repetitively and the participant rated their size on a discrete scale. There was no good or bad response thus there was no feedback. Our interest was objective certainty of *individual judgment* therefore we processed the data individually. Subsuming the ratings of participants would only have given insights on the certainty of their *collective judgment*, and we leave this topic to the specialists of votes, public opinion and related issues.

This experiment differed from previous studies about certainty in several aspects. First, it studied objective certainty independent of accuracy, whereas the literature is centered on confidence (subjective certainty) *and* accuracy. Our characterization of certainty was also novel. We first characterized consistency by means of the frequency distributions of the ratings. Then we determined *cores of certainty*, i.e. regions of absolutely consistent ratings, and regions of inconsistent ratings called *peripheries*. This dichotomy provided a simple representation of the geometry of certainty. Finally, we tried to identify in cores and boundaries classical results in perceptual judgment (see next subsection). Note that in the literature on judgment, consistency is generally considered an outcome rather than an object of study (although the tendency to be consistent is sometimes considered an explanatory factor [7]). In contrast, is this experiment consistency is *the* dependent variable that allows us to establish maps of absolute certainty. The factors that may affect consistency are viewed as controlled or random parameters.

### Categorical visual judgment

The experiment presented here is a *categorical visual judgment task*. The rationale is to map some physical feature (in a broad sense) into a discrete scale of ratings. This is a particular case of perceptual rating. In a commonly accepted view, perceptual rating is the combination of two mappings. 1) A noisy perception process [8], [9] transforms perceived features (in a broad sense) into subjective magnitudes, in a covert psychological space. 2) Subjective magnitudes are transformed into ratings. When the perceived feature is mono-dimensional, it is possible to establish a *rating curve* (also called *psychophysical mapping*) between stimuli and ratings. The rating curve follows a power law with an exponent close to one for a variety of stimuli and rating modalities [10] including 2D-computer objects [11] like those used in our study.

In *categorical judgment*, there are fewer ratings than stimuli (otherwise, the task is called *absolute identification*). The compound effect of noisy perception and the reduction of dimension between stimuli and ratings is *indeterminism*, i.e., the relationship is not one-to-one. Each rating corresponds to *distributions* of intensities whose *centers* are on the rating curve. For instance, a simulation of the categorical judgment model of [12] shows that the category of a rating has a central plateau (a core) surrounded by peripheries in which the frequency of the rating decreases. As the dispersion of the perceptual noise increases, the peripheries extend and the core disappears. However, simple models and simulations should at best be interpreted as metaphors, and the reality is far more complex.

*Anchoring effects* tend to make judgment more consistent at the extremity of the range of stimuli than in the center [13], [14]. Conversely, *Bow effects* make judgment inconsistent and slow in the center of the range [15]. The *typicality* of the stimuli (representative of their rating or not) affects reaction times [16], [17]. Also, the connotation of the labels of the scale may ‘distort’ judgment [18], [19], [20]. Finally, several results challenge the view that judgments can be repeated “in identical circumstances”. The set of stimuli, the order of presentation, the rating scale and the feedback may affect judgment (*frequency effects*, [21], *primacy effects* [7], *assimilation and contrast effects* [22], [23]). In the study of consistency, these factors have to be controlled or at least documented.

### Categories, cores and peripheries

**Figure 1:**
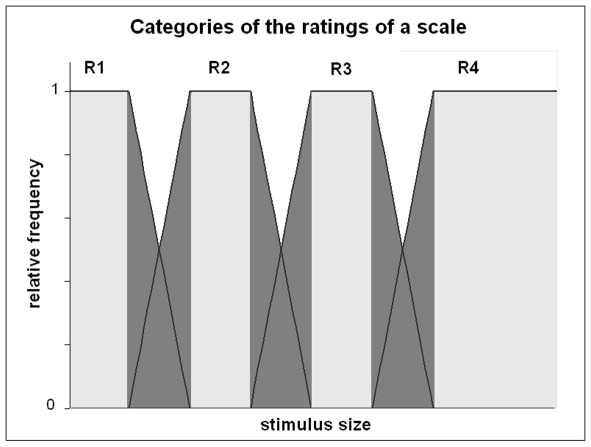
Categories of ratings (idealized). Each rating R1 … R4 corresponds to a curve with a central core where the relative frequency is 1, and peripheries where the relative frequency decreases towards 0 (the trapezoidal curves are only illustrative). *Horizontal*: Stimulus sizes. *Vertical*: relative frequency of rating. *Light gray*: cores. *Dark gray*: peripheries.

We characterize objective certainty as follows. We determine the relative frequencies of the ratings ‘R_1_’ ‘R_2_’ … ‘R_n_’ for each size ‘S’. We determine the category of each rating ‘R’ as the distribution of relative frequencies of ‘R’. These distributions of relative frequencies can be viewed as ‘likelihood’ functions, or ‘backwards probabilities’. However, for simplicity, we only use the terms of ‘relative frequencies’ and ‘conditional probabilities’. Note that the opposite representation is often encountered in the literature about rating (i.e., the distribution of ratings for each stimulus size). Both representations are in fact equivalent. The distribution of ratings for a given stimulus size can be obtained from the graphical representation of Figure 1 by drawing a vertical line and measuring the height of the intersections with the category of each rating. The *support* of a category is the set of stimulus sizes that are rated ‘R’ at least once (Figure 1).The support is divided into *core* and *peripheries*. The *core* is the set of sizes where the relative frequency of ‘R’ is (almost) 1. In the cores, judgment is absolutely consistent, i.e., objectively certain. The *peripheries* are the remainder (Figure 1). The categories of the ratings provide a global landscape of consistency in the stimulus space, and cores and peripheries divide this landscape into discrete regions of certainty/uncertainty.

Cores and peripheries are borrowed from the framework of fuzzy sets [24], [25] (see next section for details). They represent additions to the classical statistical framework, so we need to justify their introduction from the viewpoint of scientific parsimony. First, the cores have no direct equivalent in descriptive statistics. The shape of a distribution is usually characterized by its central tendency and its higher order moments, such as the dispersion and the skew, and these statistics do not tell anything about the existence of a plateau, i.e., a core. Second, we use cores and peripheries to characterize objective certainty i.e., absolute consistency. Using descriptive statistics to describe absolute consistency would have been possible, but unduly complicated. Finally, the best justification of cores and peripheries has to be experimental: these concepts should provide insights on the rating of sizes that are in line with- but not a trivial consequence of- the scientific literature.

## Methods

### Formal definition of cores and peripheries

The Theory of Fuzzy Sets [24], [25] was initially introduced to model ill-defined (fuzzy) categories like “tall men” or “numbers much greater than one”. Whereas classical sets are binary (0: *does not belong to*; 1: *belongs to*), fuzzy sets are analog: the degree of membership can be graded between 0 and 1. The degree of membership has a straightforward equivalent in the categorization literature, namely the *typicality* of the exemplars of a category [16], [26], [27]. Fuzzy sets can be interpreted in different ways, the most common being *possibility functions*. Possibility functions are similar to probabilities but are not mutually exclusive. For instance a miniature car is a car *and* a toy, and for both categories the degree of membership (possibility) is 1. For different interpretations of Fuzzy sets see [25], [29], [30].

Here, we use a *probabilistic interpretation of fuzzy sets*. We consider the category of each rating ‘R’ as a fuzzy set, i.e. a membership function µ_R_ defined on the space of sizes, and we identify membership functions with conditional probabilities, i.e., µ_R_ (s) is p(R|s), the conditional probability of rating ‘R’ given the stimulus size ‘s’. The probabilistic interpretation of fuzzy sets offers two major benefits: i) it is compatible with the probability theory, and ii) we can obtain an experimental estimate of the membership function µ_R_ as the relative frequencies of rating ‘R’. To do so, the sequence of stimuli of an experiment is considered a random sample. As the number of stimuli increases, the relative frequencies converge towards the probability density function. This experimental estimation is impossible with possibility functions [30]. In the remainder of this paper, the term of ‘membership function’ will designate the actual distributions of relative frequencies and/or the ideal curves, i.e., the conditional probability density functions of the ratings.

#### Definition of category, core and peripheries

The cores and the peripheries are defined from a fuzzy operator known as *alpha-cut* ([28], p. 19). The alpha-cut of a membership function µ_R_ is the set of sizes for which the function is greater than or equal to alpha. We define the *support* of a category of rating ‘R’ as the interval between the extreme sizes where µ_R_(s)>0, the core as the interval between the extreme sizes that compose the 1-cut, and the *peripheries* as the difference between support and core.

These definitions deserve three comments. 1) Using intervals (instead of the raw alpha-cuts) prevents scattering of the support and the core (e.g., a random erroneous rating may split the core and/or the support into two subintervals). 2) The interval-based definition allows a simple 2-fold computation: first compute the supports of all the ratings ‘R’ then compute the cores as the intervals that belong to only one support. 3) In theory, these definitions are sensitive to noise. For simplicity, we nonetheless used them in the present study, but we verified a posteriori that they were sufficient. A robust generalization of these definitions is proposed in the Section Discussion.

### Rationale of the task

#### Stimuli

The stimuli were disks presented in the central visual field on a computer monitor (diameter between 1.7 mm and 54 mm, below 8° visual angle). We expected that the centers of mass of the categories would be linearly spaced [11]. Disks were preferred to mono-dimensional stimuli like bars because in preliminary experiments, some participants reported a ‘trick’, namely comparing mentally the bar with a labeled line representing the scale.

#### Distribution of stimulus sizes

We used 64 stimulus sizes (in the range of diameters 8–260 pixels, 4 pixels steps) so that: i) the space of sizes could be considered a quasi-continuum in relation with the number of ratings (five, see below); and, ii) it was possible to present several times the same size within a reasonable amount of time. The sizes followed a uniform density probability function, so that frequency effects did not shift the categories [21] and that sequence effects were decreased by averaging.

#### Rating scale

We used a 5-point scale because it is the smallest number for which central and extreme categories have no common peripheries. It provides three different configurations: 1) extreme categories are anchored; 2) intermediary categories have a common frontier with one extreme category and 3) the central category is presumably free of direct interference from extreme categories. Also, a 5-point scale is safely below the “magical number seven” [31]. Above this (imprecise) limit, unwanted phenomena may occur: some ratings may never be used, whereas others may overlap completely.

#### Labels

The scale was labeled with letters A to E, in *increasing* order of sizes, whereas the participants (Canadian university students) were used to the *reverse* scale (academic marks, A being the highest). In order to avoid confusions, a legend (upper left corner) showed the largest and smallest possible circles tagged with E and A respectively. This scale was suggested by participants of preliminary experiments, when asked for the “less natural way of rating sizes”. We avoided numbers and intensity qualifiers for which distortions have been previously reported [18], [19]. Indeed, even with these precautions, the semantics of the labels (in fact of *any* type of labels) may have affected the geometry of the cores (see Section Discussion).

#### Inter-trials eraser

Immediately before each stimulus, the monitor displayed concentric circles in graded tones of grey (inter-trial erasers have been previously used for auditory stimuli in [32]). The eraser was used to discourage memory-based strategies in two ways: i) ‘erase’ the visual memory of previous stimuli; ii) refresh the memory of the range of sizes before each trial (the eraser was slightly larger than the largest stimuli). The effectiveness of these concentric circles was confirmed by participants in preliminary experiments.

#### Preliminary training

The participants executed a practice session with the same stimuli and a 3-point scale that lasted typically 30 minutes. The objective was to expose the participants to the entire distribution of stimulus sizes in order to control *range effects* [21] and avoid *primacy effects* [22]. We used a 3-points scale so that participants remained unexposed to the 5-points scale used for the test. Note that even with these precautions, the training probably affected the geometry of the cores (see Section Discussion).

#### Remaining effects

The eraser did *not* eliminate sequence effects based on memories of previous judgments (assimilation with last judgment; contrast with recent judgments). We expected that these effects would be decreased by averaging the data across random, uniformly distributed sequences of stimuli. In addition, the preliminary training itself may affect judgment, under the form of ‘repisodic memories’ [33] (see Section Discussion).

### Participants and procedure

Twenty healthy young volunteers (n = 20, age = 22.3 years, σ = 1.7, 10 males, 1 left-handed), with no history of motor, neurological or perceptual deficits, with normal or corrected vision participated in this study. Participants gave written informed consent in accordance with the code of ethics of the Institut universitaire de gériatrie de Montréal (certificate 2004-0301).

The stimuli were presented on a 15” LCD screen placed at about 50 cm from the eyes. No head-support was used. The experimenter verified the posture of the participant before each trial and eventually encouraged them to correct their posture. The variation of apparent size of the target caused by the head shifts can be considered additional perceptual noise. A simple geometrical model based on the distance eye-target with lateral and axial head movement showed that the variation of visual angle of the target was about 18% for 10 cm head displacement (lateral and backwards), and 9% for 5 cm (which is typically the distance that the assistant could detect).

The task was self-paced and executed with the mouse. After clicking in a start point placed in the center of the screen, an inter-stimulus eraser (concentric circles shaded from dark to white) replaced the start point and remained visible for 0.5 s. The screen was cleared during one second then the stimulus appeared at the center of the screen as a blue disk, and five boxes containing letters A to E appeared in the lower part of the screen. The participant clicked on the desired letter. After selection, no feedback was given, the screen was cleared and the start point appeared for the next trial.

Using the mouse produced larger reaction times than keys and/or buttons, but this method is safer. In preliminary experiments, participants reported fewer self-detected errors (when they were aware that they selected an unwanted category) than with keys, presumably because the movement gave them additional time for correction. According to Fitts' Law [34], this entry method did not bias the reaction time. Fitt's law states that the time to reach a target increases logarithmically with the ratio distance/diameter as measured on a line that crosses the starting point and the target. When the targets (the labels) are placed on a line, the ratio distance/diameter remains constant whatever the angle, therefore the predicted movement time is constant.

If the mouse left the start point before the stimulus appeared, the trial was tagged ‘anticipated’. Anticipated trials were discarded from the analysis of reaction time but were considered valid for computing the distributions of relative frequencies.

Participants were instructed to click in the start point and to keep the mouse at the same position until a blue disk appeared, then to click on the letter that best described the size of the blue disk as fast and accurately as possible. They were told that the letters were ordered by increasing size, from A to E, and that the disks could be any size between a dot and the size of the largest circle of the inter-stimulus eraser.

First, participants went through a practice session of 192 trials (6 blocks of 32 trials) with 3 categories. Then they performed one short practice session of 5 trials with 5 categories. After that, they executed 10 blocks of 32 trials with 5 categories. There was a pause (the duration was determined by the participant) after each block. The whole session lasted about one and a half hours.

### Data analysis

The controlled variable was the stimulus size *s*. The dependent variables were the category number (1 … 5) and the reaction time *T* (between the appearance of the stimulus and the click). Anticipated trials (in which movement began before the appearance of the disk) were considered valid for the computation of the category *c* but they were discarded for calculating the reaction time *T*.

#### Membership functions

For each Rating ‘R’, the membership function µ_R_ (s) was estimated as the relative frequency distribution, i.e., µ_R_(s)∼f_R_(s)/ Σ_R_ f_R_(s), where the frequency distribution f_R_(s) is the number of stimuli of size *s* that were rated R. The membership functions were determined with two levels of granularity: size by size for determining the cores and peripheries, and, for the figures, by bins of four consecutive sizes (corresponding to 20 stimuli, on average). The membership functions were determined based on the individual data for each participant and the data for the group.

#### Cores and peripheries

The positions and the areas of cores and peripheries were estimated for each participant and averaged across the group. The support of each membership function µ_R_ was first determined as the interval between the extreme amplitudes where µ_R_ (s)>0; the cores were determined as the intervals pertaining to the support of only one category, and the peripheries as the intervals pertaining to the support of two or more categories.

#### Effect of region type on reaction time

A two-factor ANOVA for Region Type (n = 2, Periphery vs. Core) x Participant (n = 20, random factor) was conducted on reaction time *T.* The trials for which movement was anticipated had previously been removed (they represented less than 0.6% of the trials).

#### Verification of the method of computation of cores and peripheries

The membership functions were examined post hoc in order to verify whether this simple computation was sufficient (recall that this method can misclassify scattered fragments of the cores into the peripheries, see subsection Formal Definition of Cores and Peripheries). We searched for stimuli of the core (with relative frequency close to one) that were erroneously placed in the peripheries.

#### Descriptive statistics on frequency distributions

For each Participant and rating ‘R’, the position *P_R_*, dispersion *D_R_*, and frequency *F_R_* of the category of rating ‘R’ were determined from the frequency distribution *f_R_(s)* as the Mean, Standard deviation and Number of samples, respectively. The linearity of the mapping was measured by the correlation coefficient (Pearson *r*) between the ranks of the ratings and the positions (*P_R_*), for each Participant across Categories of ratings (n = 5), and for the whole group across the set of Participant x Category (n = 100).

## Results

### Main findings

#### Shape of the categories

**Figure 2:**
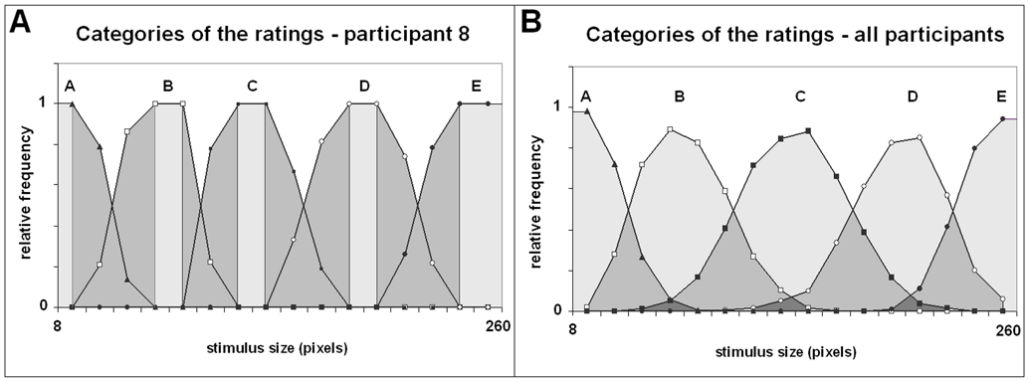
Categories of the ratings. Each category corresponds to a curve. *Horizontal*: stimulus size (diameter in pixels). *Vertical*: relative frequency. *a)* One participant. *Light gray*: cores. *Dark gray*: peripheries. *b)* Whole group. Note: the cores of central categories are reduced to a single peak.

Figure 2 depicts the categories (distributions of relative frequencies) for one participant and for the group. The whole set of curves can be found at: http://visualcategories.googlepages.com/. The group categories were roughly bell-shaped, which is compatible with the predictions of a Thurstonian model of absolute judgment, when the noise of the discriminal processes is important (for group distributions, inter-individual differences play the role of a noise). Recall that group categories would only bring information about certainty of collective judgment.

#### Individual categories presented cores and peripheries

**Figure 3:**
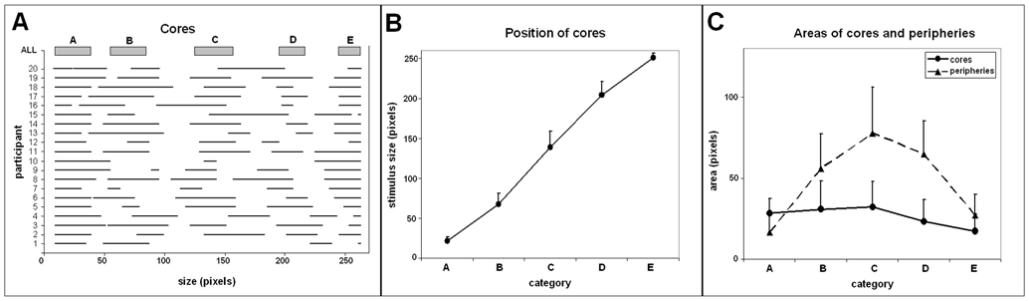
Positions and areas of cores and peripheries. Cores and peripheries are computed separately for each participant. *a)* Cores. *Horizontal*: stimulus size (diameter in pixels). *Vertical*: participants. *Note*: for participants 1 & 10, some cores are missing. *Thick horizontal bars*: ‘average core’ determined from the average position of the individual interval bounds. *b)* Position of the centers of the cores (average of the position of the individual cores). *Vertical bars*: standard deviation. *Vertical*: stimulus sizes (diameter in pixels). *Horizontal*: categories. *c)* Areas of the cores and peripheries (average of the individual areas). Common peripheries are counted twice, one for each neighbor category.

In contrast with the group categories, 94% of the individual categories had a central plateau, i.e., a core (Figure 3.a). Only 3 cores out of 20×5 = 100 categories were reduced to a single peak, and 3 cores were missing (central category ‘C’ for one participant; intermediate categories B', ‘D’, for another participant).

#### Robustness of the method of computation of cores and peripheries

None of the categories presented misclassification, i.e., fragments of the cores erroneously classified in the peripheries. This validated a posteriori the method used to compute the cores and peripheries.

#### Cores and peripheries represented similar areas

Figure 3 depicts the distributions of the positions and areas of the cores and peripheries calculated separately for each participant. Cores and peripheries had almost equivalent areas, 45.1% and 54.9% of the stimulus sizes, respectively (calculated by averaging the areas of core and peripheries of each participant). This difference is not significant (ANOVA with factor Core (1/0) and dependent variable Area: F(1, 38) = 0.613, N.S.). Most of the time, the peripheries pertained to two neighbor categories. Only 0.7% of the range of stimulus sizes pertained to three categories (this means that some category is totally covered by the neighbor categories).

#### The reaction time was longer in the peripheries than in the cores

**Figure 4:**
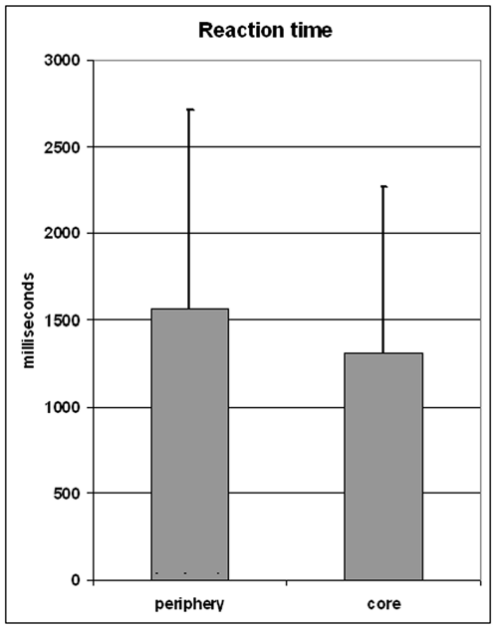
Effect of region on reaction time. *Vertical*: reaction time in milliseconds. *Horizontal*: peripheries vs. cores. *Thick bars:* Mean reaction time, determined for the whole group. *Thin bars*: standard error.

When the effects of Region type (Core vs. Periphery) and Participant (random factor) on the Reaction Time were examined, we found a significant effect of Region type, F(1, 19) = 15.6, p<.001. The reaction time was about 20% longer in peripheries than in cores (Figure 4). There were also a significant effect of Participant, F(19, 19) = 8.7, p<.0005, and a significant interaction for Region type x Participant, F(19, 6322) = 11.7, p<.0005.

### Geometry of cores and boundaries

#### The positions of the cores were not linear

The consecutive cores were not equidistant (Figure 3.b), as showed by an ANOVA on the Distance between consecutive cores (calculated from the center of mass of the cores) and factor Category (n = 4, the first category was not considered), F(3, 76) = 3.9, p<.005. The pair of cores (‘A’–‘B’ and ‘D’–‘E’) were closer than the pairs ‘B’–‘C’ and ‘C’–‘D’ (about 18% of the range of size vs. about 26%, as shown by post hoc analyses; Dunnett T3). However note that these quantitative estimates must be handled with care, given that Levene's test indicated non-homogeneous variances for the variable Distance.

#### The area of the cores decreased along the scale

The areas of the cores was relatively constant for categories ‘A’, ‘B’, and ‘C’ (about 11.7% of the range of stimulus sizes) and decreased for categories ‘D’, and ‘E’ (6.7%) (Figure 3.c). This was confirmed by an ANOVA with the factor Category (n = 5), F(4, 95) = 3.9, p<.005. Post hoc analyses (Dunnett T3; variance was not homogeneous according to Levene's test) showed that the area was significantly higher for categories ‘A’, ‘B’ and ‘C’ than for categories ‘D’, and ‘E’. The other differences were non-significant.

#### The areas of the peripheries presented a bow effect

The areas of the peripheries presented a marked bow effect, i.e., the area was maximal for the central category ‘C’ (29.8% of the range of stimulus sizes) and minimal for extreme categories ‘A’ and ‘E’. The bow effect was confirmed by an ANOVA with the factor Category (n = 5), F(4, 95) = 32.7, p<.0005. Post hoc analyses (Dunnett T3; variance was not homogeneous according to Levene's test) showed that the areas of the extreme categories (‘A’, ‘E’) were significantly lower than the areas of the intermediary categories (‘B’, ‘D’), which in turn were significantly lower than the area of the central category (‘C’). There were also significant differences between the areas of the extreme categories (‘A’<‘E’) and between the areas of the intermediary categories (‘B’<‘D’).

#### Unlike the cores, the positions of the entire distributions of relative frequencies were linear

**Figure 5:**
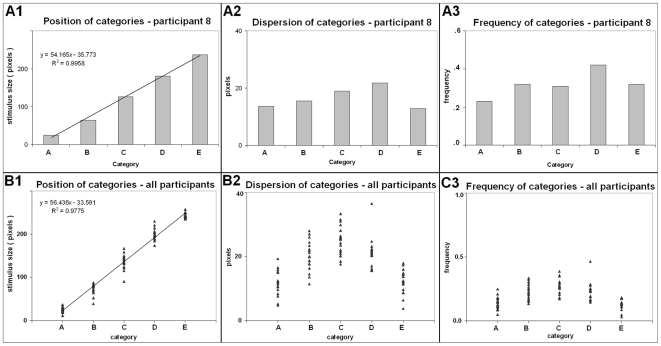
Descriptive statistics of the categories of the ratings. *From left to right*: 1) position (center of mass), 2) dispersion (standard deviation), 3) frequency of the rating (across all stimulus sizes). *Horizontal*: categories. *Vertical*: stimulus size (diameter in pixels) for position and dispersion, number of stimuli for frequency. *A:* One participant. *B:* Whole group. Scatter plots, each dot represents a Category x Participant combination.

When the entire distributions of relative frequencies were considered, the categories were linearly spaced, i.e., the positions of the centers of mass of the distributions of relative frequencies were equidistant in the range of stimulus sizes (Figure 5). This was confirmed by the high correlation coefficient obtained for individual categories (r = 0.989, n = 100, i.e., 20 participants ×5 categories, p<.01). This result is compatible with the literature, but it also means that the peripheries occulted the non-linearity of the positions of the core.

#### The rating curve was almost linear, indistinguishable from a power law

As a consequence of the former result, the rating curve (extrapolated from the centers of mass of the distributions of relative frequencies of the ratings) was almost linear. However, with only five points, correlation coefficients obtained with linear and power functions are virtually indistinguishable. Therefore the rating curves are compatible with the power law with an exponent close to one consistently reported in the literature.

## Discussion

### Cores were ubiquitous

The individual categories of the ratings almost always presented a core. We can reasonably conclude that the presence of the cores reflect a real phenomenon, namely the existence of a region of consistent individual judgment for each rating. Several factors support the view that the cores were not experimental artifacts. i) The definition of the cores and the computation method were minimalist and required no arbitrary thresholds and/or parameters. ii) The task was not particularly easy (so that the presence of cores would have been mandatory), or at least it is more difficult than classical discrimination tasks using two categories. iii) The consistency of judgment was not manipulated by any type of feedback. The preliminary training with a 3-point scale indeed provided *repisodic memories* (subsumed memories of sequences of episodes) that may have facilitated (or biased) categorical judgments [33]. However, *all* the categories of the 5-point scale had cores, even those that were absent from the 3-point scale. Finally, the significant difference in reaction time between cores and peripheries indicates a difference in the judgment process, probably due to competing decisions in the peripheries [17]. Indeed, this could be explained only in terms of consistency, but this also shows that the dichotomy cores-peripheries is not unrealistic.

### Wide peripheries

The peripheries represented about half of the range of stimulus sizes (54%). This may result from several experimental factors. i) The small inter-stimulus spacing increased the effects of perception noise. ii) The mono-dimensional, artificial stimuli may be more difficult to classify (i.e., choose between concurrent ratings) than familiar, multidimensional stimuli like faces, which produce relatively crisp categories [35]. iii) The lack of feedback and the self-determined categories may difficult classification. This is has to be contrasted with the crisp frontiers obtained when categories are perceptual, i.e., direct outcome of perception, like phonemes [36], and when the feedback is designed to increase the discrimination at the periphery [37].

### Peripheries vs. number of categories?

A remarkable fact is that during the training with a 3-point scale, the peripheries represented 27% of the range of sizes, vs. 54% in the experiment. Although we cannot compare quantitatively training and task, it is remarkable that the area of the peripheries was roughly proportional to the number of frontiers (3-point scale: 2 frontiers, 27%; 5-point scale: 4 frontiers, 54%). This provides a working hypothesis for future research, namely that until information processing limitations occur, the width of the peripheries in categorical judgment is coarsely constant.

### Unexpected geometry of certainty

The first unexpected result is that the area of the cores decreased along the scale. The most likely explanation is the perceptual noise: the imprecision on the subjective magnitude of visual stimuli (the ‘noise’) is roughly proportional to the stimulus size [10]. This imprecision makes the ratings increasingly inconsistent as their size increases, which ‘erodes’ the cores more and more towards the upper extremity of the scale. A second unexpected result was the non-linear placement of the cores, which appeared to be closer from the extremities of the scale than expected. This may result from anchoring effects: the imprecision is different at the two extremities of a core (lower towards the anchor) and the apparent shift may result from this difference of ‘erosion’. However, an equally plausible explanation is that the training with the 3-points scales enlarged the central category thus shifting the intermediate categories towards the extremities. Further experiments are thus required to determine whether this non-linearity is reproducible, and in this case, to determine its causes.

### Expected results

The categories (centers of mass of the distributions) were linearly spaced as expected from the evidence on the rating curve [11]. Note that the non-linear position of the cores was invisible at the level of the distribution. Whatever the cause of the phenomenon, this shows conclusively than classical descriptive statistics do not capture adequately consistency and objective certainty. The peripheries presented a bow effect, i.e., they were wider towards the center of the scale. This is compatible with the bow effects reported in absolute identification for the reaction times, the imprecision (rate of errors) and the dispersion of the ratings [15], [38]. However, in the case of certainty, the bow effect may have a simpler explanation: namely that the periphery is one-sided for the extreme categories and two-sided for central categories.

### Individual vs. collective judgment

The cores were only observed on individual data. In group data, they did not appear. The immediate consequence is that most of the results discussed here would have been invisible on group data. The mechanical explanation is that inter-individual differences played the role of a ‘noise’ with an order of magnitude greater than individual imprecision, i.e., all the stimuli had a different rating at some point, thus the cores were void. It is worth underlining that group data represent *collective* judgment, and it is clear that the objective certainty of collective judgment requires a smoother definition.

### How noise affects the cores

There is an inverse relationship between noise and cores. In the present case, the fist source of noise was perception (including the variability of visual angle due to small head shifts). This type of noise increases the probability of inconsistent judgments, thus it tends to ‘erode’ the cores, given that the cores are regions of *absolute* consistency. This effect mostly occurs at the frontiers (in the center of a category, the rating remains the same even if the perceived size fluctuates). A second source of noise may be random erroneous judgments (e.g., selecting involuntarily the wrong response). Such errors may occur in the middle of the categories and as the number of stimuli increases they may scatter the cores. The first symptom of scattering will be the presence of misclassified elements (of the cores into the peripheries and/or vice-versa). In the present case, we verified a posteriori that there was no trace of scattering. However, other experimental setups may be more ‘noisy’, and the issue of noise should be dealt with.

### Computing cores in presence of noise

For simplicity (or ‘methodological parsimony’) we suggest using in the first place the basic definition of the cores and the simple computation method presented here. If the data set shows traces of scattering, more complex methods should be considered. In this case, we suggest using a simple noise-resistant technique based on a generalization of the original definition of the cores. Instead of 1-cuts, the cores may be defined as α-cuts (i.e., relative frequencies > = α are in the core), which provides tolerance to a given proportion of random errors. Then, the threshold α may be adjusted automatically to the data set, so that the resulting cores are stable, e.g., by means of the plateaus method [39]. Automatic adjustment of parameters is strongly recommended, because manually-adjusted parameters decrease the significance of the results, just like arbitrary assumptions decrease the significance of a theory. Indeed, the field of signal processing offers a variety of techniques to cancel noise, threshold adjustment being only one of them. In any case, the important point is to assess the robustness of the technique a posteriori on the actual data set, or a priori, e.g., by means of simulations on artificial data sets randomly generated from preliminary data [47].

### Fuzzy tools, but no fuzzy framework

Cores and peripheries are borrowed from the theory of fuzzy sets, and they depict a geometry that is remarkably similar to the ideal membership functions used in fuzzy paradigms [28], [40]. However, in the present study, cores and peripheries were determined within the classical statistical framework. We did not assume the axiomatic of Fuzzy Sets or their canonical interpretations in terms of typicality or possibility [24], [29]. Also we did not use Fuzzy Sets to model the internal structure of categories [16], [41] or to model perception and judgment processes [42], [43]. Cores and peripheries were used to describe schematically the data and were by no means explanatory concepts. Although of considerable interest, a fuzzy model of certainty was beyond the scope of this study.

### The internal structure of categories

The internal structure (or internal representation) of categories is a useful construct to explain and/or describe the observations in Categorization and Category Learning. There exist alternative theories, e.g., prototype-based, exemplars-based, frontier-based, rule-based [44], [45]. However, a ubiquitous, theory-independent observation is that categories are generally graded [46]. It is tempting to link certainty with the internal structure of the categories of the ratings, e.g., to identify certainty with the typicality [16], [26], [27]. However, this would be unfounded. Typicality and confidence (subjective certainty) are different concepts, and in categorization tasks, both seem to participate in the *apparent* gradedness (see discussion in [6]). A second obstacle is that the internal structure is covert and difficult to extrapolate from the literature, because it depends on the way categories were defined to- and learned by- participants [44]. We thus preferred to remain empirical, and we leave to others the challenge to establish relationships between the internal structure of the categories of the ratings and certainty.

### Further studies on certainty in categorization and judgment

The present study was primarily a proof of concept. It showed that objective certainty can be documented experimentally. This study revealed unexpected phenomena, i.e., ubiquitous presence of cores, the shift of the cores towards the extremities of the scale, the decrease of the cores along the scale. These phenomena provide a complement to well-documented effects in the field of perceptual judgment (perception noise, decision noise, bow and anchoring effect, variation of resolution with stimulus intensity). However, future experiments on certainty in categorization and judgment have to account for the factors that affect the cores, like the perceptual noise (e.g., the spacing of stimuli), information processing limitations (number of ratings) or simply the factors that were insufficiently controlled in the present study, like the categories used in the preliminary training and the labels of the scale.

### Further studies on objective vs. subjective certainty

The paradigm of the present study was the objective certainty of individual judgments, without any consideration of accuracy. Previous works on confidence (i.e., subjective certainty), over- and under-confidence have been conducted in relation with accuracy. For instance in perception tasks, the common modality was comparative judgment, for which accuracy is clearly defined [4]. However, confidence is to some extent independent of accuracy, possibly related to domain knowledge independent of any specific responses [48], to individual factors (but not with cognitive styles [49]) and/or to the familiarity with stimuli [50]. The protocol presented here suggests interesting possibilities for the comparison of objective and subjective certainty (assessed by means of questionnaires) without accuracy constraints, in a task that is free from domain knowledge and that uses connotation-free stimuli. This line of study may provide insights into the mutual relationships between confidence, consistency of judgment and the resultant objective certainty.

## References

[1] Hornby AS (2005). Oxford Advancer Learner's Dictionary of Current English.. http://www.oup.com/elt/catalogue/teachersites/oald7/on.

[2] Harvey N (1997). Confidence in judgment.. Trends in Cognitive Sciences.

[3] Gonzàlez-Vallejo C, Bonham A (2007). Aligning confidence with accuracy: Revisiting the role of feedback.. Acta Psychologica.

[4] Olsson H, Juslin P (2000). The sensory sampling model: theoretical developments and empirical findings.. Food Quality and Preference.

[5] Kvidera S, Koutstaal W (2008). Confidence and Decision Type Under Matched Stimulus Conditions: Overconfidence in Perceptual but Not Conceptual Decisions.. Journal of Behavioral Decision Making.

[6] Estes Z (2004). Confidence and gradedness in semantic categorization: Definitely somewhat artifactual, maybe absolutely natural.. Psychonomic Bulletin and Review.

[7] Haubensak G (1992). The consistency model: A process model for absolute judgments.. Journal of Experimental Psychology: Human Perception and Performance.

[8] Fechner GT, Rand B (1860–1912). Elements of psychophysics, XII and XVI. Translated by HS Langfeld.. The Classical Psychologists (1912).

[9] Thurstone LL (1927). A law of comparative judgment.. Psychological Review.

[10] Stevens SS (1957). On the psychophysical law.. Psychological Review.

[11] Spence I (1990). Visual psychophysics of simple graphical elements.. Journal of Experimental Psychology: Human Perception and Performance.

[12] Braida LD, Durlach N (1972). Intensity perception. II. Resolution in one-interval paradigms.. Journal of the Acoustical Society of America.

[13] Marley AAJ, Cook VT (1984). A fixed rehearsal capacity interpretation of limits on absolute identification performance.. British Journal of Mathematical and Statistical Psychology.

[14] Petrov AA, Anderson JR (2005). The dynamics of scaling: A memory-based anchor model of category rating and absolute identification.. Psychological Review.

[15] Lacouture Y (1997). Bow, range, and sequential effects in absolute identification: A response-time analysis.. Psychological Research.

[16] McCloskey ME, Glucksberg S (1978). Natural categories: Well defined or fuzzy sets?. Memory and Cognition.

[17] Dale R, Kehoe C, Spivey MJ (2007). Graded motor responses in the time course of categorizing atypical exemplars.. Memory and Cognition.

[18] Smith J, Kaufman H, Baldasare J (1984). Direct estimation considered within a comparative judgment framework.. American Journal of Psychology.

[19] Bartoshuk LM, Duffy VB, Green BG, Hoffman HJ, Ko C-W (2004). Valid across-group comparisons with labeled scales: The gLMS versus magnitude matching.. Physiology & Behavior.

[20] Stevens SS (1958). Adaptation-level vs. the relativity of judgment.. The American Journal of Psychology.

[21] Parducci A (1965). Category judgments: A range-frequency model.. Psychological Review.

[22] Lockhead GR (2004). Absolute judgments are relative: A reinterpretation of some psychophysical ideas.. Review of General Psychology.

[23] Ward LM (1972). Category judgments of loudness in the absence of an experimenter-induced identification function: Sequential effects and power function fit.. Journal of Experimental Psychology.

[24] Zadeh LA (1965). Fuzzy sets.. Information & Control.

[25] Zadeh LA (2005). Toward a generalized theory of uncertainty (GTU): An outline.. Information Sciences.

[26] Rosch EH, Moore T (1973). On the internal structure of perceptual and semantic categories.. Cognitive development and the acquisition of language.

[27] Shoben EJ, Wilson TL (1998). Categorization in judgments of relative magnitude.. Journal of Memory and Language.

[28] Klir GJ, Yuan B (1995). Fuzzy sets and fuzzy logic – Theory and applications..

[29] Bilgiç T, Turksen IB, Dubois D, Prade H (1999). Measurement of membership functions: Theoretical and empirical work.. Handbook of fuzzy sets and systems, vol. 1, Fundamentals of fuzzy sets.

[30] Kandel A, Byatt WJ (1978). Fuzzy sets, fuzzy algebra, and fuzzy statistics.. Proceedings of the IEEE.

[31] Miller G (1956). The magical number seven – plus or minus two: Some limits on our capacity for processing information.. Psychological review.

[32] Jesteadt W, Luce RD, Green DM (1977). Sequential effects in judgments of loudness.. Journal of Experimental Psychology: Human Perception and Performance.

[33] Ward LM (1987). Remembrance of sounds past: Memory and psychophysical scaling.. Journal of Experimental Psychology : Human Perception and Performance.

[34] Fitts PM (1954). The information capacity of the human motor system in controlling the amplitude of movement.. Journal of Experimental Psychology.

[35] Beale JM, Keil FC (1995). Categorical effects in the perception of faces.. Cognition.

[36] Pastore RE, Harnad S (1987). Categorical perception: Some psychophysical models.. Categorical perception: The groundwork of cognition.

[37] Goldstone RL (1994). Influences of categorization on perceptual discrimination.. Journal of Experimental Psychology: General.

[38] Lacouture Y, Marley AAJ (1995). A Mapping Model of Bow Effects in Absolute Identification.. Journal of Mathematical Psychology.

[39] Fimbel EJ, Perez Domingo P, Lamoureux D, Beuter A (2005). Automatic detection of movement disorders using recordings of rapid alternating movements.. Journal of Neurosciences Methods.

[40] Norwitch AM, Turksen IB (1984). A model for the measurement of membership and the consequences of its empirical implementation.. Fuzzy Sets and Systems.

[41] Hersh H, Caramazza A (1976). A fuzzy set approach to modifiers and vagueness in natural language.. Journal of Experimental Psychology: General.

[42] Ellison JW, Massaro DW (1997). Featural evaluation, integration and judgment of facial affect.. Journal of Experimental Psychology: Human Perception and Performance.

[43] Ward LM, Armstrong J, Golestani N (1996). Intensity resolution and subjective magnitude in psychophysical scaling.. Perception & Psychophysics.

[44] Ashby FG, Maddox WT (2005). Human category learning.. Annual Review of Psychology.

[45] Hampton J (1995). Similarity-based categorization: the development of prototype theory.. Psychologica Belgica.

[46] Barsalou L, Neisser U (1987). The instability of graded structure: Implications for the nature of concepts.. Concepts and conceptual development: Ecological and intellectual factors in categorization.

[47] Wong W, Norwich KH (1997). Simulation of human sensory performance.. Biosystems.

[48] Koriat A (2008). When confidence in a choice is independent of which choice is made.. Psychonomic Bulletin and Review.

[49] Blais AR, Thompson MM, Baranski JV (2005). Individual differences in decision processing and confidence judgments in comparative judgment tasks: The role of cognitive styles.. Personality and Individual Differences.

[50] Estes Z (2003). Domain differences in the structure of artifactual and natural categories.. Memory and Cognition.

